# Fetal Exposure to Chinese Famine Increases Obesity Risk in Adulthood

**DOI:** 10.3390/ijerph17103649

**Published:** 2020-05-22

**Authors:** Chao Song, Meng Wang, Zheng Chen, Yecheng Yao, Ganyu Feng, Yanning Ma, Jing Fan, Ailing Liu

**Affiliations:** National Institute for Nutrition and Health, Chinese Center for Disease Control and Prevention, 27 Nanwei Road, Xicheng District, Beijing 100050, China; songchao@ninh.chinacdc.cn (C.S.); wangmeng19930317@hotmail.com (M.W.); chenzheng@ninh.chinacdc.cn (Z.C.); yaoyc@ninh.chinacdc.cn (Y.Y.); fenggy@ninh.chinacdc.cn (G.F.); mayn@ninh.chinacdc.cn (Y.M.); jing_zwtcheroyl@163.com (J.F.)

**Keywords:** famine, obesity, fetal life

## Abstract

Fetal exposure to famine may have long-term consequences in adulthood. The purpose of the present study was to explore the association between famine exposure in fetal life (Chinese famine in 1959–1961) and obesity risk in adulthood. A total of 8054 subjects (3422 male, 4632 female) were recruited from the cross-sectional 2010–2012 China National Nutrition and Health Survey (CNNHS). The subjects born in 1960 and 1961 were selected as the exposed group, while the subjects born in 1963 were selected as the unexposed group. Multiple linear or logistic regression was performed to examine the association between fetal exposure to famine and risk of obesity (body mass index (BMI), waist circumference (WC), obesity, central obesity) adjusting for gender, education level, economic status, physical exercise, sedentary time, smoking, drinking, the intake of livestock and poultry and the intake of cereal and beans. Compared with the unexposed group, WC increased by 0.52 cm after adjusting the covariates *(p =* 0.021) and females in the exposed group had a significantly higher prevalence of central obesity with an odds ratio (OR) of 1.15 (1.01,1.31) after adjusting the confounders (*p* = 0.030). WC increased by 0.71 cm, 1.21 cm after adjusting the covariates compared with the unexposed group among the total subjects and the female subjects in urban areas (*p* = 0.021, *p* = 0.001). The female subjects had a significantly higher prevalence of obesity and central obesity, with ORs of 1.34 (1.04,1.71) (*p* = 0.022), 1.28 (1.07,1.53) (*p* = 0.008) respectively. Our results suggest that fetal exposure to the Chinese famine increased obesity risk in adulthood, and the association was stronger in female and urban subjects.

## 1. Introduction

Overweight and obesity have received major attention worldwide. One study presented that the combined prevalence of overweight and obesity rose by 27.5% for adults between 1980 and 2013, and the number of overweight and obese individuals increased from 857 million in 1980 to 2.1 billion in 2013 [1]. Another study showed a total of 603.7 million adults were obese in 2015, and the prevalence of obesity has doubled in 73 countries and has continuously increased in most other countries since 1980 [2]. In China, the prevalence of overweight, obesity and central obesity increased significantly with a secular trend among both men and women [3]. The 2010–2012 China National Nutrition and Health Survey (CNNHS) results showed the prevalence of overweight and obesity amongst residents aged 18 years and older were 30.1% and 11.9%, respectively [4]. Moreover, a previous study showed that overweight and obesity were estimated to cause 3.4 million deaths globally in 2010 [2]. Overweight and obesity also imposed a substantial economic burden in China, which accounted for 42.9% of the medical and non-medical yearly cost of the major non-communicable diseases (NCDs) at 90.768 billion RMB [5]. Overweight and obesity are becoming a significant economic burden and a serious public health problem worldwide. Epidemiological studies showed that overweight and obesity were risk factors of diseases such as diabetes, hypertension, dyslipidemia, cardiovascular disease and cancer [6]. Central obesity is related to some NCDs independently of overall obesity, and higher waist circumference (WC) was positively associated with higher mortality at all levels of body mass index (BMI) from 20 to 50 kg/m^2^, even for those in the normal BMI range [7,8,9]. Obesity has many risk factors, such as genes, diet, and lifestyle [6].

Increasing evidence has indicated that nutrition status in early life has a long-term impact on chronic diseases in adulthood. Fetal undernutrition lead to metabolic and structural changes which have been found beneficial for early survival, but might increase some diseases in adulthood [10,11,12,13,14,15,16,17,18,19,20,21,22,23,24,25,26,27,28,29,30,31,32,33,34,35]. The Chinese famine from 1959 to 1961 is considered to be one of the largest and most severe famines, which provides an opportunity to explore the association between fetal famine exposure and health outcomes. Previous studies indicated that exposure to famine during early life may increase obesity, hypertension, diabetes and other diseases in adulthood [10,11,12,13,16,19,20,22,23,24,25,26,28,30,32,33,36,37,38,39,40,41,42]. Regarding obesity, some studies focused their attention on the fetal exposure to famine and BMI or WC in some regions of China or foreign countries [13,19,20,30,31,38,42]. In the present study, we utilized the national data of CNNHS in 2010–2012 to confirm the associations of famine exposure in fetal life with obesity indicators including obesity, BMI, WC and central obesity in adulthood.

## 2. Materials and Methods

### 2.1. Study Design

The CNNHS was a nationally representative cross-sectional study conducted by the National Institute for Nutrition and Health, Chinese Center for Disease Control and Prevention (NINH, China CDC). The 2010–2012 survey covered all regions throughout China except for Taiwan, Hong Kong, and Macao. The country was divided into four strata: big cities, medium and small cities, ordinary rural areas and poor rural areas by economy and social level. A total of 150 surveillance sites were selected, and in each site six villages/communities (75 households were then randomly sampled from each village/community) were randomly selected. Questionnaires were used to collect information on the demographic characteristics. Height was measured using a stadiometer after removing shoes, body weight was measured with light clothes using a beam scale and WC was measured using a waist circumference tape only after breathing out. The accuracy of the height, weight and WC were 0.1 cm, 0.1 kg and 0.1 cm, respectively. All anthropometric measurement staff were trained according to the standard procedure [43].

The Chinese famine lasted for three years in 1959–1961. There was data on the mortality rate in China (1956–62) that showed that the mortality rate in 1959–61 was higher than that in 1956–58, and in 1962 the mortality decreased to a lower level [26]. Therefore, we established our famine cohort: the subjects born in 1960 and 1961 were selected as the famine exposed group, whereas subjects born in 1963 were selected as the unexposed group. The subjects who had suffered from liver/kidney/heart diseases/cancer were excluded from our study, and finally 8054 subjects (4206 for the exposed group, 3848 for the unexposed group) were analyzed. The protocol of “Fetal origin hypothesis of diabetes: thrifty genotype hypothesis or thrifty phenotype” was approved by the Ethical Committee of NINH, China CDC (2013–010). Signed consent forms were obtained from all subjects.

### 2.2. Assessment of Variables

Information about demographic characteristics, dietary factors, smoking and drinking status, exercise data and anthropometric data were derived from the questionnaires by trained investigators. Self-reported education levels were classified as illiteracy to primary school, junior middle school, senior high school and higher. Current economic status was assessed by the per capita annual income of households in 2011, and was divided into three levels: low, middle and high. Smoking, drinking and physical exercise in leisure time were classified as “yes” or “no”. The sedentary time included the time spent watching TV, using computers, playing video games and reading in leisure time.

A validated semi-quantitative food frequency questionnaire and a 24-h recall method for the last 3 consecutive days (2 weekdays and 1 weekend day) were used to collect the dietary intake. In the present study, we only considered the intake of cereal and beans and the intake of livestock and poultry as confounders, as they had been found to associate with obesity [44,45,46]. The cereal and beans intake level was divided into insufficient (<40 g/d), sufficient (≥40 g/d, ≤75 g/d) and excessive (>75 g/d) based on the “Dietary guideline for Chinese residents (2016)” [47]. The livestock and poultry intake level was divided into insufficient (<50 g/d), sufficient (≥50 g/d, ≤150 g/d) and excessive (>150 g/d).

BMI was calculated as the weight in kilograms divided by height in meters squared (kg/m^2^). According to the criterion of weight for adults (WST 428-2013) [48], we defined underweight as BMI < 18.5, normal as BMI 18.5–23.9, overweight as BMI 24.0–27.9 and obesity as BMI ≥ 28.0. Central obesity was defined as WC 90 cm or more in men and 85 cm or more in women.

### 2.3. Statistical Analysis

The statistical software package SAS version 9.4 (SAS Institute, Cary, NC, USA) was used for data analysis. Chi-squared and nonparametric tests were used for the comparison of difference between the exposed and unexposed groups. We used multiple linear or logistic regression to estimate the association between fetal exposure to famine and risk of obesity indicators (BMI, WC, obesity, central obesity) adjusting for gender, education level, economic status, physical exercise, sedentary time, smoking, drinking, the intake of livestock and poultry and the intake of cereal and beans. Model 1 was adjusted for gender, education level and economic status. Model 2 was adjusted for the variables in Model 1 and some lifestyle factors including smoking, drinking, physical exercise and sedentary time. Model 3 was adjusted for the variables in Model 2 and dietary factors including cereal and beans intake as well as livestock and poultry intake.

## 3. Results

### 3.1. Characteristics of the Subjects

A total of 8054 subjects (3422 male, 4632 female) were included in the present study, with the median age of 49.6 years. General characteristics of participants between the exposed and unexposed groups are shown in Table 1. Except for age, education level, economic status, cereal and beans intake level and WC, the difference of basic characteristics between the two groups was not significant.

### 3.2. Associations between Fetal Famine Exposure and Obesity Risk

Table 2 presents the associations of fetal famine exposure with obesity risk. Significant associations were found among the total subjects and the female subjects. Compared with the unexposed group, WC in all subjects increased by 0.53 cm and 0.52 cm before and after adjusting all the covariates (*p* = 0.019, *p* = 0.021), which was consistent with the results among female subjects. Females in the exposed group had a significantly higher prevalence of central obesity with odds ratios (ORs) of 1.16(1.02,1.31), 1.16(1.02,1.32) and 1.15(1.01,1.31) in Model 1, Model 2 and Model 3 respectively. No other significant difference of the remaining indicators was observed in unadjusted and adjusted models. We did not find significant results among the male subjects.

### 3.3. Associations between Fetal Famine Exposure and Obesity Risk in Different Areas

**Figure 1:**
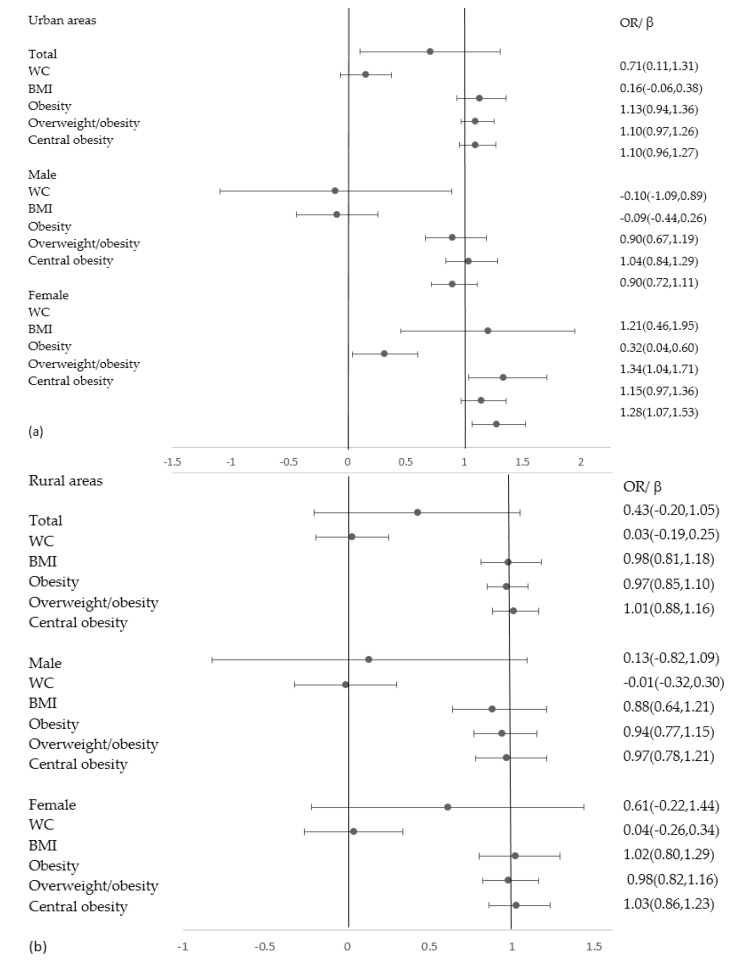
Subgroup analyses of the association between fetal famine exposure and obesity risk adjusting confounders. (**a**) Associations between fetal famine exposure and obesity risk in urban areas; (**b**) Associations between fetal famine exposure and obesity risk in rural areas.

Table 3 and Figure 1 present the associations of fetal famine exposure with obesity risk in different areas. Significant associations were found among the total subjects and the female subjects in urban areas. Compared with the unexposed group, WC increased by 0.71 cm after adjusting all the covariates (*p* = 0.021). The increased WC was significantly associated with the fetal famine exposure before and after adjusting for the confounding factors in females (*p* = 0.006, *p* = 0.001). Compared with the unexposed group, the female subjects had a significantly higher prevalence of obesity and central obesity with ORs of 1.34(1.04,1.71) (*p* = 0.022) and 1.28(1.07,1.53) (*p* = 0.008). We did not find significant results between fetal famine exposure and obesity risk among the male subjects.

## 4. Discussion

Our results suggest that fetal exposure to the Chinese famine increased obesity risk in adulthood based on the national samples. A study among 1339 adults conducted in Nigeria indicated that fetal-infant exposure to famine was associated with WC and overweight as compared to people born after the famine [31]. Results from 741 people in Amsterdam showed that maternal malnutrition during early gestation was associated with higher BMI and WC in 50-year-old women [38]. Liu et al. used the data from two population-based cross-sectional surveys conducted in Qingdao, China, which presented that exposure to famine in early life was associated with increased risks of obesity, and the indicator was BMI [20]. Wang et al. investigated the association between early nutritional status during the famine and the risk of overweight and obesity in adulthood in a Chongqing Chinese population, and found the Great Chinese Famine led to overweight in females [30]. Liu et al. evaluated the risk of central obesity among the subjects born during 1956–1961 and compared it with that of participants born during 1962–1964 using WC as the indicator [19]. Meng et al. found that famine exposure during early life might increase risks of overweight and obesity in females based on the data of China Kadoorie Biobank [37]. Huang et al. found that postnatal exposure during the first 2–3 years of life increased BMI, and exposure during pregnancy and infancy reduced BMI based on the data of women born in 1957–1963 [13]. Although most of the previous studies conducted in China and foreign countries have examined similar associations, the data in this present study were derived from a large, nationally representative surveillance of the Chinese population. The Great Chinese Famine in 1959–1961 is one of the most disastrous catastrophes, so we analyzed the subjects born in 1960–1961 and 1963. Compared with foreign famine studies, the Chinese famine lasted much longer and affected more people, which provided us with the opportunity to examine the famine exposure and later outcomes in adulthood.

The association was stronger in female subjects, which was consistent with some previous studies [37,38,49,50]. One study showed that exposure to famine during gestation had an effect on the sex ratio of live-born babies, and the percentage of boys born alive was lower [39], which may have led to the stronger association in females than that in males. Two cross-sectional surveys conducted in Qingdao, China also showed that famine exposure in early life was associated with an increased risk of metabolic syndrome in later life, especially in women [36]. The subjects in our study were about 50 years old when the 2010–2012 CNNHS was conducted, and our results were similar to a study conducted in Amsterdam, which found maternal malnutrition during early gestation was associated with higher BMI and WC in 50-year-old women but not in men. A study in western Netherlands also found that maternal undernutrition may lead to increased adiposity later in life in female offspring, which due to famine exposure in utero can lead to various adverse metabolic or mental phenotypes, depending on the sex of the exposed individual [50,51].

In the present study, the areas were classified into urban and rural areas based on residence, not on birthplace. We performed an areas-stratified analysis, and the only significant association was found in urban areas, not in rural areas. As urbanization happened throughout China in the last century, the results did not represent the prevalence of obesity in the early 1960s. According to the proposals referred by Gluckman, people currently live in evolutionarily novel environments, and the mismatch between the evolved physiological capabilities and contemporary exposures can lead to ill health. This mismatch is especially relevant to food preferences and consumption and to energy expenditure, which have changed greatly over several decades in some countries undergoing socioeconomic improvement [52,53,54]. In the past few decades, China has been undergoing a rapid social transition, and modernization has led to great lifestyle changes and increasing risks for chronic diseases, especially compared with the period of famine. Thus, we deduced that the findings may have been due to the mismatch. In other words, the subjects in our study experienced the famine in 1959–1961, and in their adulthood the environment made great progress, so their food preference and lifestyle changed. Compared with the rural residents, the environment of the urban residents changed dramatically and the disparity amongst urban residents was more apparent.

The current study indicates that exposure to famine during the prenatal period may be a risk factor of obesity, and our sample came from a large national survey, which covered the majority of the regions throughout China. We considered some lifestyle factors, including smoking, drinking, diet and physical activity. However, the present study also has some limitations, namely, that the subjects of child exposure to famine were not included and the nutrition status of childhood was not collected, as our study is a retrospective study.

## 5. Conclusions

Our results confirmed that fetal exposure to famine was associated with obesity risk in adulthood and the association was stronger in female and urban subjects. The data suggest gender-specific and long-term consequences of maternal undernutrition.

## Figures and Tables

**Table 1:** Characteristics of the subjects.

	Total	Unexposed	Exposed	*p-*Value
Total	8054	3848	4206	
Gender				0.393
Male	3422(42.5%)	1616(42.0%)	1806(42.9%)	
Female	4632(57.5%)	2232(58.0%)	2400(57.1%)	
Age	49.6(48.5,50.9)	48.4(47.8,49.1)	50.9(50.2,51.6)	**<0.001**
Education level				**<0.001**
Illiteracy to primary school	2408(29.9%)	1108(28.8%)	1300(30.9%)	
Junior middle school	3123(38.8%)	1668(43.3%)	1455(34.6%)	
Senior high school and higher	2523(31.3%)	1072(27.9%)	1451(34.5%)	
Economic status				**0.026**
Low	3806(47.3%)	1800(46.8%)	2006(47.7%)	
Middle	2957(36.7%)	1448(37.6%)	1509(35.9%)	
High	815(10.1%)	357(9.3%)	458(10.9%)	
Unknown	476(5.9%)			
Smoking				0.155
No	5515(68.5%)	2675(69.5%)	2840(67.5%)	
Yes	2521(31.3%)	1165(30.3%)	1356(32.2%)	
Unknown	18(0.2%)			
Drinking				0.120
No	5086(63.1%)	2462(64.0%)	2624(62.4%)	
Yes	2951(36.6%)	1381(35.9%)	1570(37.3%)	
Unknown	17(0.2%)			
Physical exercise				0.154
No	1804(91.0%)	909(91.7%)	895(90.3%)	
Yes	164(8.3%)	74(7.5%)	90(9.1%)	
Sedentary time	2.0(2.0,3.0)	2.0(2.0,3.0)	2.0(2.0,3.0)	0.234
Cereal and beans intake level				**0.012**
Insufficient	4853(60.3%)	2369(61.6%)	2484(59.1%)	
Sufficient	583(7.2%)	264(6.9%)	319(7.6%)	
Excessive	160(2.0%)	60(1.6%)	100(2.4%)	
Unknown	2458(30.5%)			
Livestock and poultry intake level				0.326
Insufficient	1942(24.1%)	914(23.8%)	1028(24.4%)	
Sufficient	1257(15.6%)	596(15.5%)	661(15.7%)	
Excessive	2397(29.8%)	1183(30.7%)	1214(28.9%)	
Unknown	2458(30.5%)			
WC (cm)	82.2(75.8,89.1)	82.0(75.4,89.0)	82.5(76.0,89.3)	**0.012**
BMI (kg/m^2^)	24.1(22.0,26.5)	24.1(22.0,26.5)	24.2(22.0,26.5)	0.477
Obesity				0.956
Underweight	202(2.5%)	100(2.6%)	102(2.4%)	
Normal	3534(43.9%)	1692(44.0%)	1842(43.8%)	
Overweight	2948(36.6%)	1405(36.5%)	1543(36.7%)	
Obesity	1064(13.2%)	501(13.0%)	563(13.4%)	
Unknown	306(3.8%)			
Central obesity				0.597
No	5229(64.9%)	2517(65.4%)	2712(64.5%)	
Yes	2522(31.3%)	1184(30.8%)	1338(31.8%)	
Unknown	303(3.8%)			

Data are presented as median (P25, P75) for continuous variables and N(%) for categorical variables. *p-*Values in *t*-test for difference in means or χ^2^ test for difference in proportions between exposed and unexposed group. Bold numbers mean significant results.

**Table 2:** Associations between fetal famine exposure and obesity risk.

	Unexposed	Exposed			
		Unadjusted	Model 1	Model 2	Model 3
Total					
WC					
β	1.00 (Ref)	**0.53(0.09,0.97)**	**0.51(0.07,0.94)**	**0.55(0.11,0.99)**	**0.52(0.08,0.96)**
*p*-Value		**0.019**	**0.023**	**0.014**	**0.021**
BMI					
β	1.00 (Ref)	0.06(−0.09,0.22)	0.08(−0.08,0.23)	0.09(−0.06,0.25)	0.08(−0.07,0.24)
*p*-Value		0.412	0.328	0.242	0.291
Obesity					
ORs(95%CI)	1.00 (Ref)	1.04(0.91,1.18)	1.05(0.92,1.20)	1.06(0.92,1.20)	1.05(0.92,1.20)
*p*-Value		0.591	0.453	0.423	0.438
Overweight/obesity					
ORs(95%CI)	1.00 (Ref)	1.01(0.93,1.11)	1.02(0.93,1.11)	1.02(0.93,1.12)	1.02(0.93,1.11)
*p*-Value		0.748	0.739	0.644	0.729
Central obesity					
ORs(95%CI)	1.00 (Ref)	1.05(0.96,1.16)	1.06(0.96,1.16)	1.06(0.96,1.17)	1.05(0.95,1.16)
*p*-Value		0.299	0.264	0.229	0.308
Male					
WC					
β	1.00 (Ref)	0.04(−0.66,0.75)	−0.02(−0.72,0.68)	−0.00(−0.70,0.69)	−0.03(−0.73,0.67)
*p-*Value		0.904	0.953	0.989	0.928
BMI					
β	1.00 (Ref)	−0.03(−0.27,0.21)	−0.05(−0.28,0.18)	−0.05(−0.29,0.18)	−0.06(−0.30,0.17)
*p*-Value		0.802	0.671	0.661	0.607
Obesity					
ORs(95%CI)	1.00 (Ref)	0.90(0.73,1.11)	0.90(0.73,1.11)	0.90(0.73,1.11)	0.90(0.73,1.11)
*p*-Value		0.330	0.324	0.332	0.322
Overweight/obesity					
ORs(95%CI)	1.00 (Ref)	1.01(0.87,1.16)	0.99(0.86,1.14)	0.98(0.85,1.14)	0.98(0.85,1.13)
*p*-Value		0.930	0.860	0.820	0.765
Central obesity					
ORs(95%CI)	1.00 (Ref)	0.96(0.82,1.11)	0.94(0.81,1.09)	0.93(0.80,1.09)	0.93(0.79,1.08)
*p*-Value		0.548	0.411	0.393	0.335
Female					
WC					
β	1.00 (Ref)	**0.79(0.24,1.34)**	**0.92(0.37,1.47)**	**0.97(0.41,1.53)**	**0.93(0.37,1.49)**
*p*-Value		**0.005**	**0.001**	**<0.001**	**0.001**
BMI					
β	1.00 (Ref)	0.14(−0.07,0.34)	0.18(−0.02,0.38)	0.20(−0.01,0.41)	0.19(−0.02,0.40)
*p*-Value		0.183	0.085	0.058	0.072
Obesity					
ORs(95%CI)	1.00 (Ref)	1.14(0.96,1.35)	1.17(0.99,1.38)	1.17(0.98,1.38)	1.16(0.98,1.38)
*p*-Value		0.132	0.072	0.079	0.082
Overweight/obesity					
ORs(95%CI)	1.00 (Ref)	1.02(0.91,1.15)	1.04(0.93,1.18)	1.05(0.93,1.19)	1.05(0.93,1.18)
*p*-Value		0.694	0.478	0.402	0.457
Central obesity					
ORs(95%CI)	1.00 (Ref)	1.13(0.99,1.28)	**1.16(1.02,1.31)**	**1.16(1.02,1.32)**	**1.15(1.01,1.31)**
*p*-Value		0.061	**0.024**	**0.020**	**0.030**

Data are presented as β for the increasing value of WC and BMI, ORs (95%CI) for the risk odds ratio (95% confidence interval) of overweight, obesity and central obesity. Bold numbers mean significant results.

**Table 3:** Associations between fetal famine exposure and obesity risk in different areas.

	Unexposed	Exposed			
		Unadjusted	Model 1	Model 2	Model 3
Urban					
Total					
WC					
β	1.00 (Ref)	0.61(−0.01,1.23)	**0.69(0.09,1.28)**	**0.71(0.11,1.31)**	**0.71(0.11,1.31)**
*p*-Value		0.053	**0.023**	**0.020**	**0.021**
BMI					
β	1.00 (Ref)	0.15(−0.07,0.36)	0.16(−0.06,0.37)	0.16(−0.06,0.38)	0.16(−0.06,0.38)
*p*-Value		0.188	0.154	0.145	0.150
Obesity					
ORs(95%CI)	1.00 (Ref)	1.11(0.92,1.33)	1.12(0.93,1.35)	1.13(0.94,1.36)	1.13(0.94,1.36)
*p*-Value		0.273	0.224	0.202	0.207
Overweight/obesity					
ORs(95%CI)	1.00 (Ref)	1.10(0.97,1.25)	1.10(0.97,1.25)	1.10(0.97,1.26)	1.10(0.97,1.26)
*p*-Value		0.143	0.147	0.137	0.137
Central obesity					
ORs(95%CI)	1.00 (Ref)	1.09(0.95,1.25)	1.10(0.96,1.26)	1.10(0.96,1.27)	1.10(0.96,1.27)
*p*-Value		0.200	0.165	0.153	0.158
Male					
WC					
β	1.00 (Ref)	0.06(−0.93,1.05)	−0.05(−1.04,0.93)	−0.09(−1.08,0.89)	−0.10(−1.09,0.89)
*p*-Value		0.901	0.919	0.854	0.849
BMI					
β	1.00 (Ref)	−0.03(−0.37,0.31)	−0.07(−0.41,0.27)	−0.09(−0.43,0.26)	−0.09(−0.44,0.26)
*p*-Value		0.865	0.688	0.623	0.607
Obesity					
ORs(95%CI)	1.00 (Ref)	0.91(0.68,1.20)	0.90(0.68,1.19)	0.90(0.68,1.20)	0.90(0.67,1.19)
*p*-Value		0.496	0.466	0.465	0.448
Overweight/obesity					
ORs(95%CI)	1.00 (Ref)	1.10(0.89,1.34)	1.06(0.86,1.30)	1.04(0.84,1.28)	1.04(0.84,1.29)
*p*-Value		0.383	0.596	0.707	0.705
Central obesity					
ORs(95%CI)	1.00 (Ref)	0.94(0.76,1.15)	0.91(0.74,1.13)	0.90(0.72,1.11)	0.90(0.72,1.11)
*p*-Value		0.532	0.398	0.317	0.324
Female					
WC					
β	1.00 (Ref)	**1.04(0.30,1.78)**	**1.15(0.41,1.88)**	**1.20(0.46,1.95)**	**1.21(0.46,1.95)**
*p*-Value		**0.006**	**0.002**	**0.002**	**0.001**
BMI					
β	1.00 (Ref)	0.26(−0.01,0.54)	**0.30(0.02,0.58)**	**0.32(0.04,0.60)**	**0.32(0.04,0.60)**
*p*-Value		0.063	**0.033**	**0.027**	**0.025**
Obesity					
ORs(95%CI)	1.00 (Ref)	**1.29(1.01,1.65)**	**1.31(1.03,1.68)**	**1.33(1.04,1.71)**	**1.34(1.04,1.71)**
*p*-Value		**0.042**	**0.029**	**0.023**	**0.022**
Overweight/obesity					
ORs(95%CI)	1.00 (Ref)	1.11(0.94,1.30)	1.13(0.95,1.33)	1.14(0.97,1.35)	1.15(0.97,1.36)
*p*-Value		0.228	0.160	0.119	0.109
Central obesity					
ORs(95%CI)	1.00 (Ref)	**1.23(1.03,1.46)**	**1.26(1.05,1.50)**	**1.28(1.07,1.53)**	**1.28(1.07,1.53)**
*p*-Value		**0.024**	**0.013**	**0.008**	**0.008**
Rural					
Total					
WC					
β	1.00 (Ref)	0.40(−0.22,1.01)	0.39(−0.23,1.01)	0.49(−0.13,1.12)	0.43(−0.20,1.05)
*p*-Value		0.207	0.213	0.121	0.182
BMI					
β	1.00 (Ref)	−0.03(−0.25,0.18)	0.01(−0.21,0.22)	0.05(−0.17,0.27)	0.03(−0.19,0.25)
*p*-Value		0.773	0.963	0.659	0.776
Obesity					
ORs(95%CI)	1.00 (Ref)	0.95(0.79,1.15)	0.98(0.81,1.18)	0.99(0.82,1.19)	0.98(0.81,1.18)
*p*-Value		0.622	0.831	0.887	0.803
Overweight/obesity					
ORs(95%CI)	1.00 (Ref)	0.94(0.83,1.06)	0.96(0.84,1.09)	0.97(0.86,1.11)	0.97(0.85,1.10)
*p*-Value		0.308	0.485	0.694	0.620
Central obesity					
ORs(95%CI)	1.00 (Ref)	1.00(0.87,1.14)	1.02(0.89,1.17)	1.03(0.90,1.18)	1.01(0.88,1.16)
*p*-Value		1.000	0.787	0.673	0.878
Male					
WC					
β	1.00 (Ref)	0.10(−0.85,1.05)	0.13(−0.82,1.07)	0.23(−0.71,1.18)	0.13(−0.82,1.09)
*p*-Value		0.835	0.794	0.629	0.781
BMI					
β	1.00 (Ref)	−0.02(−0.32,0.29)	−0.02(−0.32,0.29)	0.02(−0.29,0.32)	−0.01(−0.32,0.30)
*p*-Value		0.921	0.912	0.913	0.959
Obesity					
ORs(95%CI)	1.00 (Ref)	0.88(0.65,1.20)	0.89(0.65,1.21)	0.90(0.66,1.24)	0.88(0.64,1.21)
*p*-Value		0.419	0.447	0.533	0.443
Overweight/obesity					
ORs(95%CI)	1.00 (Ref)	0.94(0.77,1.13)	0.93(0.77,1.13)	0.95(0.78,1.16)	0.94(0.77,1.15)
*p*-Value		0.497	0.477	0.625	0.551
Central obesity					
ORs(95%CI)	1.00 (Ref)	0.97(0.79,1.20)	0.97(0.78,1.20)	0.99(0.79,1.24)	0.97(0.78,1.21)
*p*-Value		0.799	0.765	0.949	0.795
Female					
WC					
β	1.00 (Ref)	0.54(−0.27,1.35)	0.62(−0.20,1.44)	0.69(−0.14,1.52)	0.61(−0.22,1.44)
*p*-Value		0.190	0.136	0.104	0.150
BMI					
β	1.00 (Ref)	0.00(−0.29,0.29)	0.02(−0.27,0.32)	0.06(−0.24,0.36)	0.04(−0.26,0.34)
*p*-Value		0.993	0.876	0.696	0.799
Obesity					
ORs(95%CI)	1.00 (Ref)	1.02(0.81,1.28)	1.04(0.82,1.31)	1.02(0.81,1.30)	1.02(0.80,1.29)
*p*-Value		0.870	0.754	0.855	0.895
Overweight/obesity					
ORs(95%CI)	1.00 (Ref)	0.96(0.81,1.13)	0.98(0.82,1.16)	0.98(0.83,1.17)	0.98(0.82,1.16)
*p*-Value		0.632	0.780	0.849	0.781
Central obesity					
ORs(95%CI)	1.00 (Ref)	1.04(0.87,1.24)	1.05(0.88,1.26)	1.05(0.88,1.25)	1.03(0.86,1.23)
*p*-Value		0.658	0.555	0.612	0.787

Data are presented as β for the increasing value of WC and BMI, and ORs (95%CI) for the risk odds ratio (95% confidence interval) of overweight, obesity and central obesity. Bold numbers mean significant results.

## References

[1] Ng M., Fleming T., Robinson M., Blake T., Nicholas G., Christopher M., Mullany E.C., Stan B., Cristiana A., Abera Semaw F. (2014). Global, regional, and national prevalence of overweight and obesity in children and adults during 1980–2013: A systematic analysis for the global burden of disease study 2013. Lancet.

[2] Collaborators G.B.D.O., Afshin A., Forouzanfar M.H., Reitsma M.B., Sur P., Estep K., Lee A., Marczak L., Mokdad A.H., Moradi-Lakeh M. (2017). Health effects of overweight and obesity in 195 countries over 25 years. N. Engl. J. Med..

[3] Xi B., Liang Y., He T., Reilly K.H., Hu Y., Wang Q., Yan Y., Mi J. (2012). Secular trends in the prevalence of general and abdominal obesity among chinese adults, 1993–2009. Obes. Rev..

[4] Bureau of Disease Prevention and Control National Health and Family Planning Commission of the PRC (2016). Report on Chinese Residents’ Chronic Diseases and Nutrition (2015).

[5] Zhang J., Shi X., Liang F. (2013). Economic costs of both overweight and obesity among chinese urban and rural residents, in 2010. Chin. J. Epidemiol..

[6] Hruby A., Manson J.E., Qi L., Malik V.S., Rimm E.B., Sun Q., Willett W.C., Hu F.B. (2016). Determinants and consequences of obesity. Am. J. Public Health.

[7] Zhang C., Rexrode K.M., van Dam R.M., Li T.Y., Hu F.B. (2008). Abdominal obesity and the risk of all-cause, cardiovascular, and cancer mortality: Sixteen years of follow-up in us women. Circulation.

[8] Cerhan J.R., Moore S.C., Jacobs E.J., Kitahara C.M., Rosenberg P.S., Adami H.O., Ebbert J.O., English D.R., Gapstur S.M., Giles G.G. (2014). A pooled analysis of waist circumference and mortality in 650,000 adults. Mayo Clin. Proc..

[9] Snijder M.B., van Dam R.M., Visser M., Seidell J.C. (2006). What aspects of body fat are particularly hazardous and how do we measure them?. Int. J. Epidemiol..

[10] Liu L., Wang W., Sun J., Pang Z. (2018). Association of famine exposure during early life with the risk of type 2 diabetes in adulthood: A meta-analysis. Eur. J. Nutr..

[11] Roseboom T., de Rooij S., Painter R. (2006). The dutch famine and its long-term consequences for adult health. Early Hum. Dev..

[12] Shi Z., Nicholls S.J., Taylor A.W., Magliano D.J., Appleton S., Zimmet P. (2018). Early life exposure to chinese famine modifies the association between hypertension and cardiovascular disease. J. Hypertens..

[13] Huang C., Li Z., Wang M., Martorell R. (2010). Early life exposure to the 1959–1961 chinese famine has long-term health consequences. J. Nutr..

[14] Jiang X., Ma H., Wang Y., Liu Y. (2013). Early life factors and type 2 diabetes mellitus. J. Diabetes Res..

[15] Zhang W., Luan R. (2020). Early-life exposure to the chinese famine of 1959–61 and risk of hyperuricemia: Results from the china health and retirement longitudinal study. BMC Public Health.

[16] Qin L.-L., Luo B.-A., Gao F., Feng X.-L., Liu J.-H. (2020). Effect of exposure to famine during early life on risk of metabolic syndrome in adulthood: A meta-analysis. J. Diabetes Res..

[17] Klimek P., Leitner M., Kautzky-Willer A., Thurner S. (2014). Effect of fetal and infant malnutrition on metabolism in older age. Gerontology.

[18] Xin X., Wang W., Xu H., Li Z., Zhang D. (2019). Exposure to chinese famine in early life and the risk of dyslipidemia in adulthood. Eur. J. Nutr..

[19] Liu D., Yu D.-M., Zhao L.-Y., Fang H.-Y., Zhang J., Wang J.-Z., Yang Z.-Y., Zhao W.-H. (2019). Exposure to famine during early life and abdominal obesity in adulthood: Findings from the great chinese famine during 1959–1961. Nutrients.

[20] Liu L., Pang Z.C., Sun J.P., Xue B., Wang S.J., Ning F., Qiao Q. (2017). Exposure to famine in early life and the risk of obesity in adulthood in qingdao: Evidence from the 1959–1961 chinese famine. Nutr. Metab. Cardiovasc. Dis..

[21] Zong L., Cai L., Liang J., Lin W., Yao J., Huang H., Tang K., Chen L., Li L., Lin L. (2019). Exposure to famine in early life and the risk of osteoporosis in adulthood: A prospective study. Endocr. Pract..

[22] Yu C., Wang J., Li Y., Han X., Hu H., Wang F., Yuan J., Yao P., Miao X., Wei S. (2017). Exposure to the chinese famine in early life and hypertension prevalence risk in adults. J. Hypertens..

[23] Li Y., He Y., Qi L., Jaddoe V.W., Feskens E.J., Yang X., Ma G., Hu F.B. (2010). Exposure to the chinese famine in early life and the risk of hyperglycemia and type 2 diabetes in adulthood. Diabetes.

[24] Li Y., Jaddoe V.W., Qi L., He Y., Lai J., Wang J., Zhang J., Hu Y., Ding E.L., Yang X. (2011). Exposure to the chinese famine in early life and the risk of hypertension in adulthood. J. Hypertens..

[25] Li C., Lumey L.H. (2017). Exposure to the chinese famine of 1959–61 in early life and long-term health conditions: A systematic review and meta-analysis. Int. J. Epidemiol..

[26] Luo Z., Mu R., Zhang X. (2006). Famine and overweight in china. Rev. Agric. Econ..

[27] Fall C.H. (2013). Fetal malnutrition and long-term outcomes. Nestle Nutr. Inst. Workshop Ser..

[28] Hidayat K., Du X., Shi B.M., Qin L.Q. (2020). Foetal and childhood exposure to famine and the risks of cardiometabolic conditions in adulthood: A systematic review and meta-analysis of observational studies. Obes. Rev..

[29] Zheng X., Ren W., Gong L., Long J., Luo R., Wang Y. (2017). The great chinese famine exposure in early life and the risk of nonalcoholic fatty liver disease in adult women. Ann. Hepatol..

[30] Wang Y., Wang X., Kong Y., Zhang J.H., Zeng Q. (2010). The great chinese famine leads to shorter and overweight females in chongqing chinese population after 50 years. Obesity.

[31] Miranda J.J., Hult M., Tornhammar P., Ueda P., Chima C., Edstedt Bonamy A.-K., Ozumba B., Norman M. (2010). Hypertension, diabetes and overweight: Looming legacies of the biafran famine. PLoS ONE.

[32] Wang P.X., Wang J.J., Lei Y.X., Xiao L., Luo Z.C. (2012). Impact of fetal and infant exposure to the chinese great famine on the risk of hypertension in adulthood. PLoS ONE.

[33] Wang Z., Li C., Yang Z., Zou Z., Ma J. (2016). Infant exposure to chinese famine increased the risk of hypertension in adulthood: Results from the china health and retirement longitudinal study. BMC Public Health.

[34] Xu H., Li L., Zhang Z., Liu J. (2016). Is natural experiment a cure? Re-examining the long-term health effects of china’s 1959–1961 famine. Soc. Sci. Med..

[35] van Abeelen A.F., Veenendaal M.V., Painter R.C., de Rooij S.R., Dijkgraaf M.G., Bossuyt P.M., Elias S.G., Grobbee D.E., Uiterwaal C.S., Roseboom T.J. (2012). Survival effects of prenatal famine exposure. Am. J. Clin. Nutr..

[36] Ning F., Ren J., Song X., Zhang D., Liu L., Zhang L., Sun J., Zhang D., Pang Z., Qiao Q. (2019). Famine exposure in early life and risk of metabolic syndrome in adulthood: Comparisons of different metabolic syndrome definitions. J. Diabetes Res..

[37] Meng R., Si J., Lv J., Guo Y., Bian Z., Yu C., Zhou H., Tan Y., Pei P. (2016). Association between famine exposure during early life and bmi in adulthood. Chin. J. Epidemiol..

[38] Ravelli A.C., Meulen J.H.V.d., Osmond C., Barker D.J., Bleker O.P. (1999). Obesity at the age of 50 y in men and women exposed to famine prenatally. Amer. J. Clin. Nutr..

[39] Roseboom T.J., Van Der Meulen J.H., Ravelli A.C., Osmond C., Barker D.J., Bleker O.P. (2001). Effects of prenatal exposure to the dutch famine on adult disease in later life: An overview. Mol. Cell. Endocrinol..

[40] van Abeelen A.F., Elias S.G., Bossuyt P.M., Grobbee D.E., van der Schouw Y.T., Roseboom T.J., Uiterwaal C.S. (2012). Famine exposure in the young and the risk of type 2 diabetes in adulthood. Diabetes.

[41] Zhang Y., Liu X., Wang M., Song Y., Zhang L., You Y., Su Y., Liu Y., Kou C. (2018). Risk of hyperglycemia and diabetes after early-life famine exposure: A cross-sectional survey in northeastern china. Int. J. Environ. Res. Public Health.

[42] Zhou J., Zhang L., Xuan P., Fan Y., Yang L., Hu C., Bo Q., Wang G., Sheng J., Wang S. (2018). The relationship between famine exposure during early life and body mass index in adulthood: A systematic review and meta-analysis. PLoS ONE.

[43] Zhao L., Ma G., Piao J., Zhang J., Yu D., He Y., Huo J., Hu X., Yang Z., Yang X. (2016). Scheme of the 2010-2012 chinese nutrition and health surveillance. Chin. J. Prev. Med..

[44] Shu L., Zheng P.-F., Zhang X.-Y., Si C.-J., Yu X.-L., Gao W., Zhang L., Liao D. (2015). Association between dietary patterns and the indicators of obesity among chinese: A cross-sectional study. Nutrients.

[45] Zou Y., Zhang R., Xia S., Huang L., Meng J., Fang Y., Ding G. (2017). Dietary patterns and obesity among chinese adults: Results from a household-based cross-sectional study. Int. J. Environ. Res. Public Health.

[46] Cho S.S., Qi L., Fahey G.C., Klurfeld D.M. (2013). Consumption of cereal fiber, mixtures of whole grains and bran, and whole grains and risk reduction in type 2 diabetes, obesity, and cardiovascular disease. Am. J. Clin. Nutr..

[47] The Chinese Nutrition Society (2016). Dietary Guideline for Chinese Residents(2016).

[48] National Health Commission of the Family Planning Commission of the PRC (2013). CN-WS. WS/T 428-2013 Criteria of Weight for Adults; China. http://g.wanfangdata.com.cn/details/detail.do?_type=standards&id=WS/T428-2013.

[49] Yang Z., Zhao W., Zhang X., Mu R., Zhai Y., Kong L., Chen C. (2008). Impact of famine during pregnancy and infancy on health in adulthood. Obes. Rev..

[50] Stein A.D., Kahn H.S., Rundle A., Zybert P.A., van der Pal–de Bruin K., Lumey L.H. (2007). Anthropometric measures in middle age after exposure to famine during gestation: Evidence from the dutch famine. Am. J. Clin. Nutr..

[51] Tobi E.W., Lumey L.H., Talens R.P., Kremer D., Putter H., Stein A.D., Slagboom P.E., Heijmans B.T. (2009). DNA methylation differences after exposure to prenatal famine are common and timing- and sex-specific. Hum. Mol. Genet..

[52] Gluckman P.D., Hanson M.A. (2004). Living with the past: Evolution, development, and patterns of disease. Science.

[53] Gluckman P.D., Hanson M.A., Bateson P., Beedle A.S., Law C.M., Bhutta Z.Q.A., Anokhin K.V., Bougnères P., Chandak G.R., Dasgupta P. (2009). Towards a new developmental synthesis: Adaptive developmental plasticity and human disease. Lancet.

[54] Gluckman P.D., Hanson M.A., Cooper C., Thornburg K.L. (2008). Effect of in utero and early-life conditions on adult health and disease. N. Engl. J. Med..

